# Association and Validation of Yield-Favored Alleles in Chinese Cultivars of Common Wheat (*Triticumaestivum* L.)

**DOI:** 10.1371/journal.pone.0130029

**Published:** 2015-06-11

**Authors:** Jie Guo, Chenyang Hao, Yong Zhang, Boqiao Zhang, Xiaoming Cheng, Lin Qin, Tian Li, Weiping Shi, Xiaoping Chang, Ruilian Jing, Wuyun Yang, Wenjing Hu, Xueyong Zhang, Shunhe Cheng

**Affiliations:** 1 National Key Laboratory of Crop Genetics and Germplasm Enhancement, Nanjing Agricultural University, Nanjing, Jiangsu, China; 2 Key Laboratory of Wheat Biology and Genetic Improvement for Low and Middle Yangtze Valley (Ministry of Agriculture), Lixiahe Agricultural Institute of Jiangsu Province, Yangzhou, Jiangsu, China; 3 Key Laboratory of Crop Gene Resources and Germplasm Enhancement, Ministry of Agriculture/Institute of Crop Science, Chinese Academy of Agricultural Sciences, Beijing, China; 4 Crop Research Institute, Sichuan Academy of Agricultural Sciences, Chengdu, Sichuan, China; Oklahoma State University, UNITED STATES

## Abstract

Common wheat is one of the most important crops in China, which is the largest producer in the world. A set of 230 cultivars was used to identify yield-related loci by association mapping. This set was tested for seven yield-related traits, viz. plant height (PH), spike length (SL), spikelet number per spike (SNPS), kernel number per spike (KNPS), thousand-kernel weight (TKW), kernel weight per spike (KWPS), and sterile spikelet number (SSN) per plant in four environments. A total of 106 simple sequence repeat (SSR) markers distributed on all 21 chromosomes were used to screen the set. Twenty-one and 19 of them were associated with KNPS and TKW, respectively. Association mapping detected 73 significant associations across 50 SSRs, and the phenotypic variation explained (*R^2^*) by the associations ranged from 1.54 to 23.93%. The associated loci were distributed on all chromosomes except 4A, 7A, and 7D. Significant and potentially new alleles were present on 8 chromosomes, namely1A, 1D, 2A, 2D, 3D, 4B, 5B, and 6B. Further analysis showed that genetic effects of associated loci were greatly influenced by association panels, and the *R^2^* of crucial loci were lower in modern cultivars than in the mini core collection, probably caused by strong selection in wheat breeding. In order to confirm the results of association analysis, yield-related favorable alleles *Xgwm135-1A_138_*, *Xgwm337-1D_186_*, *Xgwm102-2D_144_*, and *Xgwm132-6B_128_* were evaluated in a double haploid (DH) population derived from Hanxuan10 xLumai14.These favorable alleles that were validated in various populations might be valuable in breeding for high-yield.

## Introduction

Wheat is one the most important crops in the world with a total production of about 713 million tonnes in 2013 [1]. With an increasing world population it is necessary to continuously raise production mainly through higher yields. Identification of new yield-related lociis becoming increasingly important in all food crops.

Wheat yield is determined by three key factors, viz. spikes per unit area, kernel number per spike and thousand-kernel weight. Most yield-related traits in wheat are controlled by genes with low heritability [2]. Many yield-related QTLs were identified in studies of using bi-parental populations segregating for traits such as plant height [3–8], spike length [9, 10], spikelet number per spike [10–12], kernel number per spike [10, 13, 14, 15], thousand-kernel weight [10, 16, 17, 18], kernel weight per spike [10, 19] and sterile spikelet number per spike [7, 10, 20, 21]. For example, as a diagnostic marker, *Xgwm261* closely linkedto *Rht8* on 2D, plays an important role in wheat yield improvement in southern Europe [3, 4]. Although there has been progress in identification of yield-related QTL mapping based on bi-parental populations, only a relatively small part of the total phenotypic variation within a crop species is identified in a single cross [22].

Association analysis identifies trait-marker relationships based on linkage disequilibrium [23]. This method has several advantages compared to bi-parental populations, such as (1) materials used in association analysis can be existing germplasm ranging from landraces to modern varieties and advanced lines; (2) novel and superior (favorable) alleles associated with the best phenotypes can be identified and ranked for use in breeding; (3) association mapping is more efficient and cheaper than other methods [24]; and (4) the results of association mapping apply to a wider range of genetic backgrounds. For example, Sajjad et al. [25] identified six SSR loci associated with yield-related traits on chromosome 3A, explaining 10.7 to 17.3% of the yield-related phenotypic variation in 94 wheat cultivars using 39 SSRs. Among them, *Xgwm155* and *Xwmc527*, *Xcfa2134* and *Xgwm369*, *Xgwm155*, and *Xgwm369* were associated with grain yield per plant, fertile florets per spikelet, plant height, and spike length, respectively. Wang et al. [26] genotyped 531 SSR markers in the Chinese mini core wheat collection; 22 SSR loci were associated with TKW, each explaining phenotypic variation ranging from 1.56 to 21.99%. Six loci, *Xcfa2234-3A*, *Xgwm156-3B*, *Xbarc56-5A*, *Xgwm234-5B*, *Xwmc17-7A* and *Xcfa2257-7A* accounted for more than 10% of the variation. Using the same association panel Zhang et al. [27] identified 23 SSR loci significantly associated with KNPS, and reported that favorable alleles combined with additive effects. They also identified favorable alleles at the *Xwmc304-1A*, *Xgwm311-2A*, *Xcfa2234-3A*, *Xgwm2-3A*, *Xgwm131-3B*, *Xgwm156-3B*, *Xgwm2-3D*, *Xcfe273-6A* and *Xcfa2257-7A* loci with positive effects on both TKW and KNPS. However, relatively few studies in wheat have involved mapping/analysis of multiple yield-related traits based on combined bi-parental populations and association panels.

In the present study 230 diverse common wheat cultivars were genotyped at106 SSR loci prior to association analysis of data for seven yield-related traits obtained in multiple environments with the aim of identifying favorable loci or alleles. The purposes of the study were to provide insights into utilization of association study and linkage analysis to dissect the genetic basis of traits, as well as information that may be useful for future molecular breeding in the Yangtze River Valley.

## Materials and Methods

### Plant materials

The association panel of 230 wheat genotypes included 222 Chinese, 1 USA, 1 Chilean, 4 Italian, 1 Mexican and 1 Romanian cultivars. The Chinese accessions, included 39 cultivars from Jiangsu, 10 from Anhui, 6 from Hubei, 14 from Hunan, 2 from Jiangxi, 2 from Zhejiang, 5 from Fujian, 9 from Sichuan, 3 from Guizhou, 2 from Yunnan, 36 from Henan, 19 from Shandong, 6 from Gansu, 7 from Shanxi, 18 from Beijing, 8 from Hebei, 32 from Shaanxi, 3 from Heilongjiang and 1 from Qinghai (S1 Table). A biparental DH population of 150 lines from the cross Hanxuan10 xLumai14 was also used. Both parents were historically important cultivars; Hanxuan10 was released in 1966 and Lumai14 was a high-yielding cultivar during the 1990s; it has higher KNPS, TKW and yield than Hanxuan10 [28].

### Phenotyping

The cultivar panel was planted in four environments, viz. 2008 and 2009 at the Sichuan Academy of Agricultural Sciences in Chengdu (designated 08CD and 09CD, respectively), and in 2008 and 2009 at the Lixiahe Agricultural Institute of Jiangsu Province in Yangzhou (08YZ and 09YZ, respectively).

The field experiment consisted of three randomized complete blocks. Each cultivar was planted in three 133 cm rows with 40 seeds per row, and a row spacing of 25 cm. The yield-related traits PH (cm), SL (cm), SNPS, KNPS, TKW (g), KWPS (g) and SSN were measured on an average 20 plants in the middle of each plot and expressed as means.

The 150 DH lines and parents were planted in two environments, viz. 2010 and 2011 at Changping, Beijing (DH10 and DH11, respectively). The field design was three randomized complete blocks. Each cultivar was planted in two-row plots with a length of 2 m and 30 cm spacing rows. Yield-related traits included PH (cm), SL (cm), SNPS, KNPS, TKW (g) and SSN measured on 20 plants in the middle of each plot.

Mean values of yield-related traits, standard deviations, standard errors, variation coefficients (CV) and broad sense heritabilities for each environment were analyzed by IBM SPSS Statistics 21.0.0 software (http://www.brothersoft.com/ibm-spss-statistics-469577.html). The best linear unbiased predictor (BLUP) method was used to estimate mixed means of the phenotypic traits as in the association analysis [29–31].

### SSR genotyping

Genomic DNA from 10 seedling leaves of each cultivar was extracted by the CTAB method [32]. A total of 106 SSR markers distributed across all 21 chromosomes [33] were genotyped the association set and DH population. Among them, 21 and 19 markers were previously reported to be associated with KNPS [27] and TKW [26] (S3 Table), respectively. Primer sequences and annealing temperatures (S2 Table) were obtained from GrainGenes (http://archive.gramene.org/markers/) and Somers et al. [33]. An ABI 3730 Genetic Analyzer (Applied Biosystems, Foster City, CA, USA) was used to separate amplified products after purification. Fragment sizes were determined using an internal size standard (GeneScanTM-500 LIZ, Applied Biosystems). GeneMapperV3.7 software (Applied Biosystems) was used to estimate fragment sizes (http://www.appliedbiosystems.com.cn/).

### Data analysis

General parameters of genetic diversity of each SSR marker, including MAF (major allele frequency), allele number, genetic diversity and PIC (polymorphism information content) were evaluated using PowerMarker V3.25 software [34]. To reduce spurious associations, population structure of the 230 cultivars was analyzed using Structure V2.3.2 [35]. The number of presumed sub-populations (K) was set from 1 to 15 with an admixture model and correlated allelic frequencies. This process was repeated five times. For each run, burn-in and Markov Chain Monte Carlo iterations were set to 50,000 and 100,000, respectively. The number of sub-populations and the best output was determined following the ΔK method [36]. Kinship analysis was also performed using genotypic data with SPAGeDi software [37] to determine genetic covariance between individuals. Evaluation of pairwise kinship coefficients was based on Loiselle et al. [38] with 10,000 permutation tests. All negative values between individuals were then set to 0, indicating that they were less related than random individuals [39].

The MLM (mixed linear model) module with Q + K was used for association analysis between phenotypic traits and SSRs through TASSEL 2.1 software (http://www.maizegenetics.net/) [40, 41]. The phenotypic variation explained (*R* ^*2*^) for each associated locus was calculated for alleles with frequencies>5% [26, 27]. Based on phenotypic data and the kinship matrix using TASSEL 2.1 software, the heritability (*h* ^*2*^) of each trait in different environments, defined as the proportion of genetic variance over total variance, was calculated according to the formula *h* ^*2*^ = σ_a_^2^/(σ_a_^2^+σ_e_^2^) with the MLM options of no compression or re-estimation for each marker. Here, σ_a_^2^ means genetic variance, and σ_e_^2^ indicates the residual variance. Genetic effects of favorable alleles of associated loci were evaluated by multiple comparisons and ANOVA using IBM SPSS Statistics 21.0.0 (http://www.brothersoft.com/ibm-spss-statistics-469577.html).

## Results

### Phenotypic assessment

Yield-related traits for the association panel were determined over environments 08CD, 09CD, 08YZ and 09YZ. Average values of yield-related traits were calculated according to the BLUP method and a summary of parameters for the seven traits is listed in Table 1. The CV of phenotypic traits in each environment were higher than 10% with the exception of SNPS, indicating that trait values differed between cultivars. Moreover, the average *h* ^*2*^ of PH and TKW were 72.3 and 51.1%, respectively, and higher than those of other traits in all environments.

**Table 1:** Descriptive statistics for seven phenotypic traits assessed in this study.

Trait	08CD					08YZ					09CD					09YZ					Average				
	Mean±SD^a^	Min	Max	CV^b^	*h* ^*2*^ ^c^	Mean±SD	Min	Max	CV	*h* ^*2*^	Mean±SD	Min	Max	CV	*h* ^*2*^	Mean±SD	Min	Max	CV	*h* ^*2*^	Mean±SD	Min	Max	CV	*h* ^*2*^
KNPS	35.0±7.3	15.6	64.9	20.9	32.1	54.2±7.7	35.4	78.0	14.3	35.2	36.4±7.4	19.4	80.2	20.3	25.8	54.0±9.8	33.7	81.7	18.1	46.2	44.8±5.5	32.1	69.8	12.3	48.7
KWPS	1.3±0.3	0.5	3.5	26.3	34.7	2.4±0.4	1.3	3.8	17.5	31.3	1.5±0.4	0.4	3.6	26.6	19.6	2.0±0.4	1.0	3.3	21.9	45.3	1.8±0.3	1.1	2.9	14.2	37.2
PH	92.7±16.8	51.6	134.1	18.1	69.2	97.5±14.6	55.8	133.3	15.0	75.1	97.3±18.2	52.3	143.0	18.7	71.2	98.0±15.6	65.8	150.1	15.9	58.4	96.4±15.3	57.6	135.6	15.9	72.3
SL	7.9±1.1	5.2	10.7	13.7	28.7	9.8±1.3	6.3	13.9	13.2	36.7	8.4±0.9	5.8	12.4	10.9	10.5	9.7±1.3	6.5	17.1	13.8	45.6	8.9±0.9	6.6	11.6	10.0	39.9
SNPS	20.9±1.5	16.6	25.2	7.1	26.4	21.1±1.4	17.6	24.6	6.5	21.9	21.3±1.7	12.7	27.2	7.9	17.0	19.3±1.2	16.2	22.8	6.4	33.9	20.6±1.0	18.1	23.5	4.8	27.0
SSN	3.7±1.0	0.9	7.1	26.4	30.9	1.4±0.7	0.0	3.9	49.3	15.2	4.1±1.0	1.3	7.2	24.0	12.7	1.7±0.6	0.5	4.7	34.1	25.7	2.7±0.5	1.3	4.8	18.6	27.7
TKW	40.7±5.2	26.3	61.2	12.6	37.2	38.4±5.4	23.2	52.9	14.1	27.8	40.3±8.2	21.1	76.5	20.3	44.1	37.6±5.0	22.4	52.1	13.4	51.8	39.3±4.3	28.0	51.8	11.0	51.1

^a^ SD, standard deviation

^b^ CV, coefficient of variation

^c^ Broad sense heritability

### Allelic diversity

A total of 907 alleles were detected in the association panel using 106 SSR markers. MAF ranged from 0.164 to 0.987 with a mean of 0.542. Numbers of alleles per locus varied from 2 to 25 with an average of 8.6. PIC values ranged from 0.026 to 0.903, with an average of 0.552 (S3 Table). These values indicated that the association panel had a relatively high level of molecular genetic diversity.

### Genetic structure and relative kinship among cultivars

**Fig 1:**
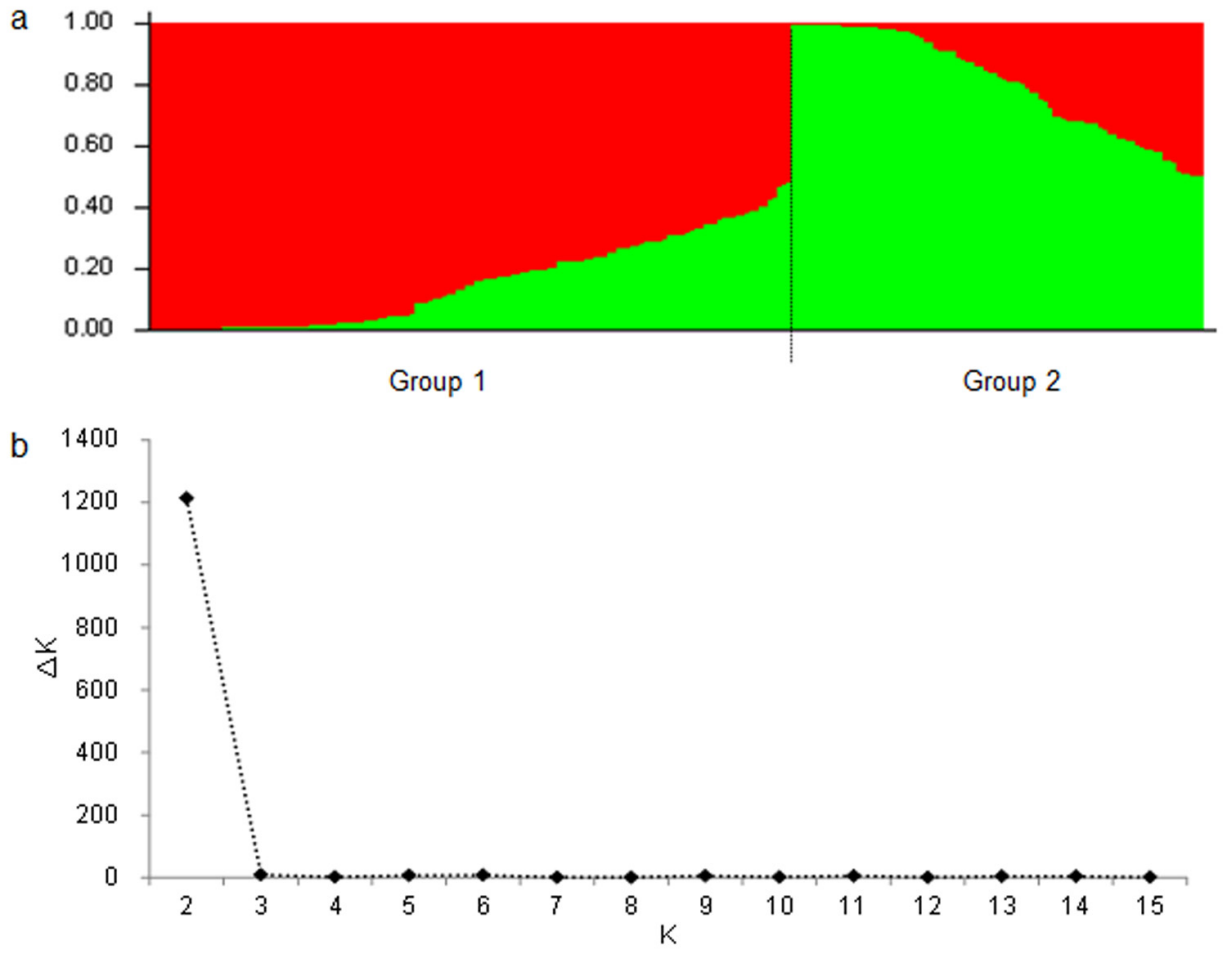
Population structure of 230 wheat cultivars based on 106 SSR markers with whole-genome coverage. a: Genetic structure produced by Structure V2.3.2; b: Number of sub-populations estimated by ΔK at a range of K values.

The population structure of the 230 cultivars was calculated based on 106 SSR markers with 907 alleles by Structure V2.3.2. The cultivars were basically divided into two sub-populations according to their geographic origins (Fig 1a). The number of presumed sub-populations (K) was set from 1 to 15 for calculating ΔK values, which reached the highest value at K = 2 (Fig 1b), confirming that the population should be divided into two sub-populations.

**Fig 2:**
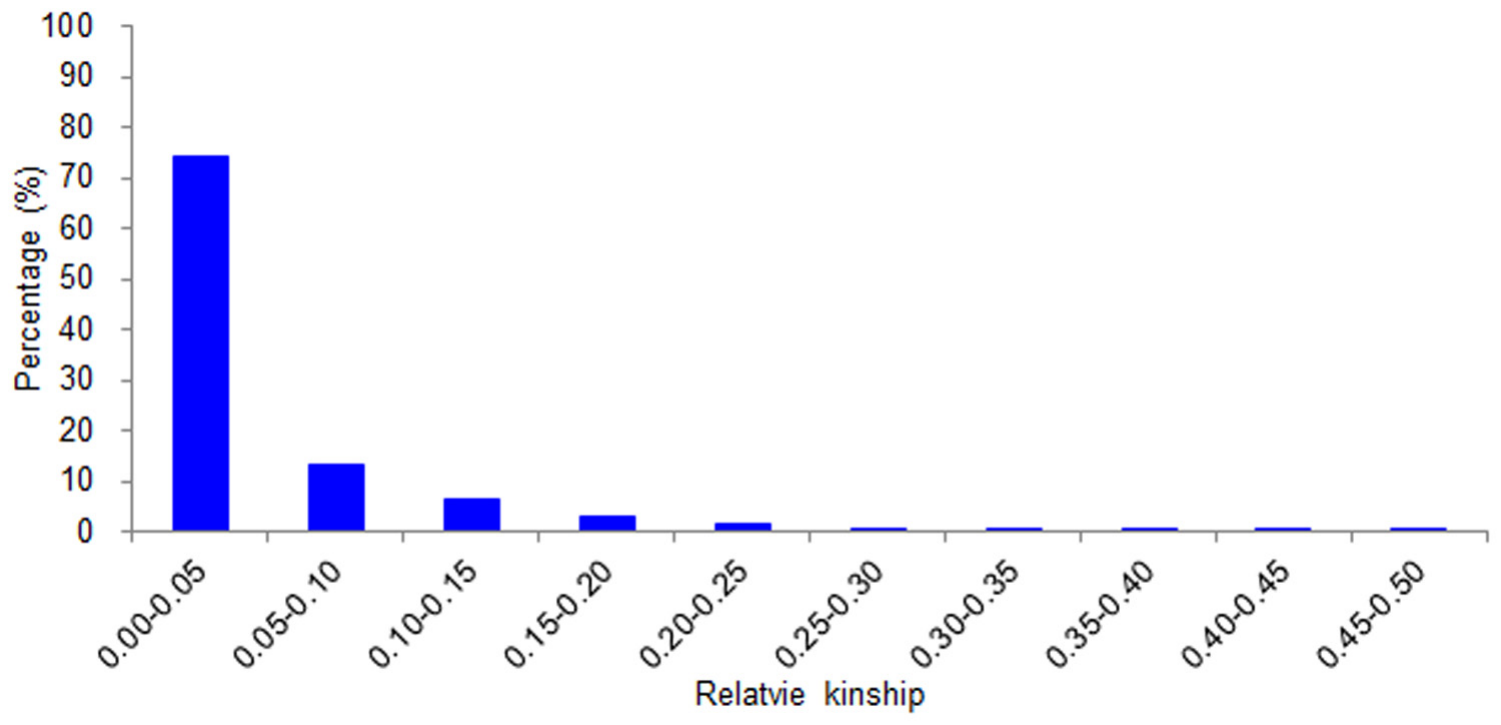
Distribution of pairwise kinship coefficients among 230 bread wheat cultivars based on 106 whole genome SSR markers.

Relative kinship coefficients between individuals were also calculated using data for 106 SSR markers (Fig 2). About 74.1% of the pairwise kinship coefficients ranged from 0 to 0.05, indicating that most cultivars had no, or only a weak, relationship with each other.

### Association analysis between seven yield-related traits and SSR markers

**Fig 3:**
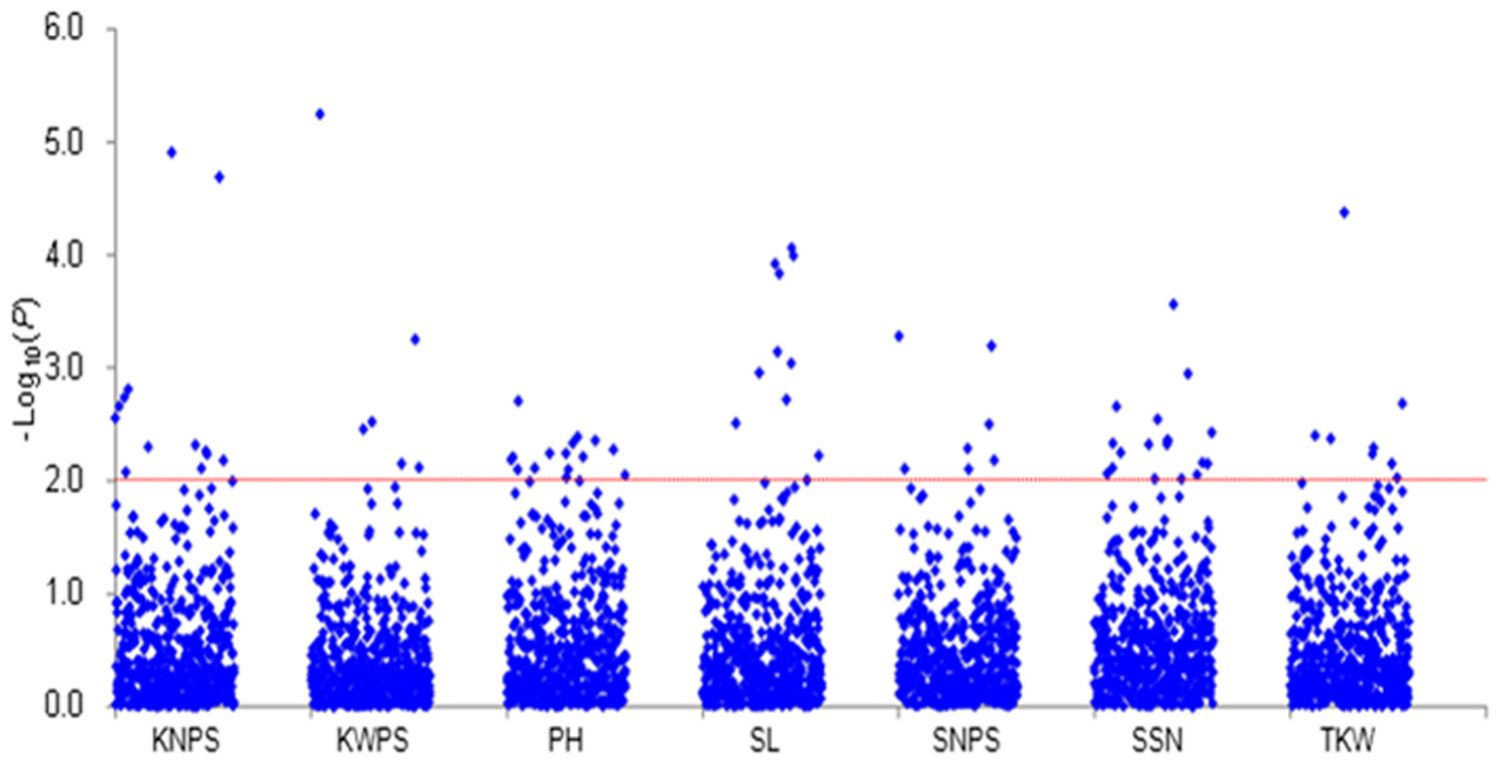
Associations of seven phenotypic traits with 106 genome-wide SSR markers illustrated as dot plots of compressed MLM at *P*<0.01. The red dotted line indicates the threshold value of significant association.

Association analysis was performed between the 907 alleles at 106 SSR loci and seven yield-related traits over four environments using a mixed linear model. Seventy three significant associations were identified at 50 SSR loci (Fig 3, Table 2) located on all chromosomes except 4A, 7A and 7D. SSR loci associated with KNPS were located on chromosome 1A, 1D, 2D, 3B, 5B, 5D and 6B, KWPS-associated loci were located on 1A, 2D and 5B, PH-associated loci were located on 1B, 2A, 2B, 3A, 4B, 5A, 5B, 6B and 7B, SL-associated loci were located on 1A, 2B, 4B, 4D, 5D and 6A, SNPS-associated loci were located on 1D, 5A, 5D, 6B and 7B, SSN-associated loci were located on 2B, 2D, 3A, 3D, 5A, 5B, 6D and 7B, and TKW-associated loci were located on 2A, 2B, 4B and 5A. In addition, seven SSR loci were significantly associated with yield-related traits in two or more environments, such as, *Xgwm135-1A* with KNPS, *Xgwm515-2A* and *Xgwm132-6B* with PH, *Xgwm219-6B* with SNPS, and *Xgwm102-2D*, *Xgwm297-7B* and *Xgwm383-3D* with SSN. Furthermore, nine SSR loci, including *Xgwm135-1A*, *Xwmc361-2B*, *Xgwm102-2D*, *Xgwm495-4B*, *Xbarc56-5A*, *Xgwm186-5A*, *Xgwm540-5B*, *Xgwm182-5D* and *Xgwm132-6B*, were significantly associated with two or more traits across environments.

**Table 2:** Seventy-three significant association signals (*P*<0.01) involving 50 SSR loci and seven yield-related traits.

Trait	Locus	Allele size (bp)	Chr.	Environment	*P* value	*R* ^*2*^	Reported by	Trait	Locus	Allele size (bp)	Chr.	Environment	*P* value	*R* ^*2*^	Reported by
KNPS	*Xgwm135*	130–150	1A	08CD	1.8×10^-3^	7.00		SL	*Xgwm194*	127–137	4D	09YZ	8.7×10^-5^	3.77	
				08YZ	5.0×10^-3^	6.67			*Xgwm495*	154–182	4B	09YZ	9.1×10^-4^	5.38	
				09CD	1.2×10^-5^	6.26			*Xgwm501*	154–182	2B	09YZ	1.9×10^-3^	5.22	
				09YZ	4.8×10^-3^	4.15			*Xgwm82*	144–150	6A	Average	6.0×10^-3^	3.14	
				Average	2.0×10^-5^	7.86		SNPS	*Xgwm219*	148–188	6B	09CD	5.1×10^-3^	12.14	
	*Xgwm102*	134–154	2D	08CD	1.6×10^-3^	18.09						09YZ	6.4×10^-4^	7.85	
				Average	6.6×10-3	17.32			*Xgwm337*	168–198	1D	08CD	5.2×10^-4^	17.08	
	*Xgwm132*	112–136	6B	08CD	2.2×10^-3^	11.98	[7]					Average	6.6×10^-3^	18.99	
	*Xgwm389*	114–142	3B	09YZ	7.7×10^-3^	19.89			*Xbarc56*	117–135	5A	08CD	7.8×10^-3^	3.08	
	*Xgwm540*	115–137	5B	09YZ	5.4×10^-3^	23.08			*Xgwm182*	156–172	5D	09YZ	3.2×10^-3^	7.88	
	*Xgwm583*	158–168	5D	09YZ	5.8×10^-3^	1.75			*Xgwm333*	146–154	7B	09CD	7.9×10^-3^	10.36	
	*Xgwm642*	174–202	1D	08CD	8.4×10^-3^	8.27		SSN	*Xgwm102*	134–154	2D	08CD	8.6×10^-3^	12.80	
	*Xwmc24*	125–145	1A	08CD	2.8×10^-3^	9.16	[41]					09CD	9.7×10^-3^	8.40	
KWPS	*Xgwm135*	130–150	1A	08CD	5.7×10^-6^	8.75						Average	6.9×10^-3^	12.26	
				Average	5.6×10^-4^	7.54			*Xgwm297*	132–168	7B	08CD	2.2×10^-3^	8.31	[7]
	*Xgwm102*	134–154	2D	Average	7.6×10^-3^	14.67						09YZ	1.1×10^-3^	3.40	
	*Xgwm484*	131–179	2D	09CD	3.0×10^-3^	15.64	[19]					Average	3.7×10^-3^	4.93	
	*Xgwm540*	115–137	5B	09YZ	7.1×10^-3^	23.93			*Xgwm383*	174–190	3D	09CD	2.8×10^-3^	1.54	
PH	*Xgwm515*	121–131	2A	08CD	2.0×10^-3^	8.89						09YZ	9.7×10^-3^	2.87	
				09CD	5.7×10^-3^	8.19			*Xgwm186*	98–150	5A	08CD	7.7×10^-3^	7.64	[45]
				Average	5.3×10^-3^	7.97						Average	7.0×10^-3^	9.39	
	*Xgwm132*	112–136	6B	08CD	6.5×10^-3^	13.57	[7]		*Xgwm55*	118–138	6D	09YZ	2.7×10^-4^	6.95	
				08YZ	7.7×10^-3^	12.21						Average	8.8×10^-3^	8.23	
	*Xgwm46*	142–188	7B	09CD	4.1×10^-3^	15.07	[43]		*Xgwm234*	225–249	5B	09CD	4.8×10^-3^	7.94	
				Average	8.8×10^-3^	15.23			*Xgwm2*	116–128	3A	09YZ	4.4×10^-3^	9.61	
	*Xgwm120*	118–162	2B	09CD	9.3×10^-3^	19.40			*Xgwm2*	220–232	3D	09YZ	4.4×10^-3^	9.66	
	*Xgwm155*	116–146	3A	09CD	8.0×10^-3^	9.89	[25]		*Xgwm415*	130–132	5A	08CD	4.6×10^-3^	7.04	
	*Xgwm186*	98–150	5A	09CD	4.7×10^-3^	7.49	[10]		*Xwmc361*	214–226	2B	09YZ	4.8×10^-3^	4.74	
	*Xgwm372*	271–331	2A	08CD	6.2×10^-3^	22.25		TKW	*Xbarc56*	117–135	5A	09CD	4.2×10^-5^	11.64	[46–49]
	*Xgwm403*	132–144	1B	09YZ	6.1×10^-3^	6.82						Average	7.1×10^-3^	2.92	
	*Xgwm495*	154–182	4B	09YZ	4.4×10^-3^	21.27			*Xgwm148*	138–166	2B	09YZ	5.1×10^-3^	8.75	
	*Xgwm540*	115–137	5B	08YZ	5.7×10^-3^	10.58						Average	9.4×10^-3^	8.42	
	*Xgwm95*	105–131	2A	08CD	7.9×10^-3^	13.12			*Xgwm495*	154–182	4B	08YZ	4.3×10^-3^	13.94	
SL	*Xgwm164*	105–128	1A	08YZ	3.1×10^-3^	8.20						Average	2.1×10^-3^	17.86	
				Average	9.8×10^-3^	7.66			*Xgwm294*	62–112	2A	09YZ	5.8×10^-3^	13.23	
	*Xgwm135*	130–150	1A	09CD	1.1×10^-3^	6.01			*Xwmc361*	214–226	2B	08YZ	4.0×10^-3^	4.68	[45]
	*Xgwm182*	156–172	5D	09YZ	1.0×10^-4^	2.25	[44]								

The phenotypic variation explained (*R* ^*2*^) in overall associations varied for different traits and SSR loci, and the *R* ^*2*^ of each association ranged from 1.54 to 23.93% with a mean of 10.00% (Table 2). Among 73 associations, 27 had *R* ^*2*^ values higher than 10%and 33 were between 5% and 10%. For example, locus *Xgwm132-6B* had an R^2^ of more than 10% for KNPS in 08CD, PH in 08CD and 08YZ, and *Xgwm102-2D* on KNPS in 08CD, SSN in 08CD and 09CD, as well as *Xgwm135-1A* on KNPS in all four environments, KWPS in 08CD with more than 5% of *R* ^*2*^.

### Genetic effects of favorable alleles

The genetic effects of favorable alleles were calculated as differences between alleles and mean values. A total of 50 associated favorable alleles were identified by comparing mean phenotypic data and different alleles using multiple comparisons for alleles with allelic frequencies>5% (Table 3). Of these, the frequencies of *Xgwm135-1A*_*138*_ on KNPS, *Xwmc361-2B*_*216*_ on TKW, *Xgwm102-2D*_*144*_ on KNPS, KWPS and SSN, *Xgwm540-5B*_*115*_ on KNPS, KWPS and PH, were higher than 50%, indicating these loci might have undergone strong selection pressure during modern breeding.

**Table 3:** Favorable alleles and effects of 50SSR loci significantly (*P*<0.01) associated with seven yield-related traits.

Trait	Locus	Chr.	Favorable allele (bp)	Freq. (%)	Allele effect					Trait	Locus	Chr.	Favorable allele (bp)	Freq. (%)	Allele effect				
					08CD	08YZ	09CD	09YZ	Average						08CD	08YZ	09CD	09YZ	Average
KNPS	*Xgwm135*	1A	138	86.67	1.14	1.19	1.84	0.89	1.05	SL	*Xgwm501*	2B	160	7.83				0.36	
	*Xwmc24*	1A	141	5.22	0.10						*Xgwm495*	4B	162	15.22				0.25	
	*Xgwm642*	1D	202	5.65	1.44						*Xgwm194*	4D	133	14.35				0.38	
	*Xgwm102*	2D	144	50.00	2.31				1.66		*Xgwm182*	5D	164	9.57				0.20	
	*Xgwm389*	3B	130	6.52				5.61			*Xgwm82*	6A	146	12.61					0.23
	*Xgwm540*	5B	115	60.43				3.85		SNPS	*Xgwm337*	1D	186	5.22	0.58				0.36
	*Xgwm583*	5D	164	5.65				1.20			*Xbarc56*	5A	119	48.26	0.09				
	*Xgwm132*	6B	112	12.17	0.98						*Xgwm182*	5D	164	9.57				0.31	
KWPS	*Xgwm135*	1A	134	5.22	0.04				0.05		*Xgwm219*	6B	186	40.43			1.00	0.75	
	*Xgwm102*	2D	144	50.00					0.08		*Xgwm333*	7B	150	16.09			0.35		
	*Xgwm484*	2D	163	13.91			0.10			SSN	*Xwmc361*	2B	218	20.00				-0.01	
	*Xgwm540*	5B	115	60.43				0.16			*Xgwm102*	2D	144	50.00	-0.19		-0.09		-0.08
	*Xgwm403*	1B	138	16.96				-3.38			*Xgwm2*	3A	128	13.91				-0.16	
PH	*Xgwm372*	2A	331	20.43	-5.01						*Xgwm2*	3D	232	13.91				-0.16	
	*Xgwm515*	2A	125	36.09	-2.52		-2.64		-2.05		*Xgwm383*	3D	188	43.04			-0.12	-0.01	
	*Xgwm95*	2A	117	36.52	-4.45						*Xgwm186*	5A	118	6.52	-0.21				-0.14
	*Xgwm120*	2B	150	10.87			-6.03				*Xgwm415*	5A	130	42.17	-0.25				
	*Xgwm155*	3A	140	11.30			-1.56				*Xgwm234*	5B	225	8.26			-0.19		
	*Xgwm495*	4B	154	6.96				-7.92			*Xgwm55*	6D	130	33.48				-0.20	-0.12
	*Xgwm186*	5A	122	13.91			-2.74				*Xgwm297*	7B	150	7.39	-0.34			-0.04	-0.16
	*Xgwm540*	5B	115	60.43		-2.53				TKW	*Xgwm294*	2A	100	9.13				1.31	
	*Xgwm132*	6B	128	40.87	-2.76	-2.56					*Xgwm148*	2B	162	11.74				2.09	1.63
	*Xgwm46*	7B	170	10.87			-2.16		-2.09		*Xwmc361*	2B	216	73.91		0.57			
	*Xgwm135*	1A	134	5.22			0.31				*Xgwm495*	4B	154	6.96		0.73			1.29
SL	*Xgwm164*	1A	120	26.96		0.19			0.14		*Xbarc56*	5A	119	48.26			-0.05		0.05

Genetic effects of favorable alleles among various loci were also evaluated in four environments (Table 3). Seven alleles showing the largest effects on different yield-related traits, included *Xgwm389-3B*_*130*_ on KNPS (5.61), *Xgwm540-5B*_*115*_ on KWPS (0.16 g), *Xgwm495-4B*_*154*_ on PH (-7.92 cm), *Xgwm194-4D*_*133*_ on SL (0.38 cm), *Xgwm219-6B*_*186*_ on SNPS (1.00), *Xgwm297-7B*_*150*_ on SSN (-0.34), and *Xgwm148-2B*_*162*_ on TKW (2.09 g). In addition, we also detected some loci associated with multiple traits, such as *Xgwm102-2D*_*144*_ having positive effects on KNPS, KWPS and SSN, and *Xgwm132-6B*_*112*_ with positive effects on KNPS and PH.

### Favorable alleles at crucial loci were not always the major ones in breeding panels

The 40 SSR loci associated with TKW and KNPS in Wang et al. [26] and Zhang et al. [27] were re-evaluated in the current population, and only three, viz. *Xbarc56-5A* with TKW, and *Xwmc24-1A* and *Xgwm132-6B* with KNPS, were significantly associated in this study. Failure to confirm most of the previously associated loci led us question the cause. We therefore used ANOVA to verify the allelic effects at these loci in the panel. Significant allelic differences were detected at 18 loci (Table 4). Favorable alleles assigned previously at 10 loci were the major alleles, including five loci associated with KNPS (*Xgwm2-3A*_*116*_, *Xgwm108-3B*_*127*_, *Xcfd64-3D*_*239*_, *Xgwm2-3D*_*220*_ and *Xcfe273-6A*_*306*_) and five loci associated with TKW (*Xgwm312-2A*_*190*_, *Xgwm372-2A*_*331*_, *Xcfa2234-3A*_*142*_, *Xcfd266-5D*_*167*_ and *Xcfa2257-7A*_*129*_). At the other eight loci, the favorable alleles assigned previously were not the major ones in the current panel. They were *Xgwm259-1B*_*102*_, *Xgwm337-1D*_*168*_, *Xgwm609-4D*_*111*_ and *Xgwm132-6B*_*112*_, which were associated with KNPS, and *Xgwm234-5B*_*227*_, *Xgwm174-5D*_*209*_, *Xwmc168-7A*_*305*_, and *Xwmc17-7A*_*180*_ associated with TKW. We also found that the *R* ^*2*^ for TKW at nine loci were lower. For example, the *R* ^*2*^ for *Xcfa2234-3A* and *Xcfa2257-7A* in the previous report were 18.20 and 21.99%, respectively [26, 27], whereas in current population they were 4.09 and 2.05%, indicating lower genetic effects in a breeding population.

**Table 4:** Comparison of genetic effects of favorable alleles between previous and current studies.

Locus	Chr.	Effect of previous favorable alleles in the present study					Alleles with highest frequency in the present study				
		Allele (bp)	Freq. (%)	Mean ± SE	*P* value	R^2^ (%)	Allele (bp)	Freq. (%)	Mean ± SE	*P* value	R^2^ (%)
*Xgwm259* ^*&*^	1B	106	14.8	41.11 ± 0.83	0***	8.21	102	41.3	45.70 ± 0.53	0.033*	2.04
		Others	85.2	44.46 ± 0.39			Others	58.7	44.13 ± 0.49		
*Xgwm337* ^*&*^	1D	178	3.5	47.12 ± 2.00	0.236	0.66	168	44.3	45.70 ± 0.53	0***	7.79
		Others	96.5	44.73 ± 0.39			Others	55.7	44.13 ± 0.49		
*Xgwm2* ^*&*^	3A	116	71.7	45.61 ± 0.38	0***	7.5					
		Others	28.3	45.09 ± 0.92							
*Xgwm108* ^*&*^	3B	127	16.5	46.49 ± 0.82	0.040*	1.92	119	52.6	45.35 ± 0.45	0.105	1.19
		Others	83.5	44.73 ± 0.39			Others	47.4	44.13 ± 0.62		
*Xcfd64* ^*&*^	3D	239	85.2	45.04 ± 0.39	0.049*	1.74					
		Others	14.8	42.83 ± 0.96							
*Xgwm2* ^*&*^	3D	220	71.3	45.61 ± 0.38	0***	7.51					
		Others	28.7	45.09 ± 0.92							
*Xgwm609* ^*&*^	4D	117	2.6	38.84 ± 1.64	0.007**	3.49	111	47.4	45.82 ± 0.54	0.002**	4.47
		Others	97.4	44.91 ± 0.38			Others	52.6	43.52 ± 0.50		
*Xcfe273* ^*&*^	6A	306	41.7	46.22 ± 0.50	0.001**	4.98	339	56.1	43.75 ± 0.50	0.001**	4.98
		Others	58.3	43.75 ± 0.50			Others	43.9	46.22 ± 0.50		
*Xgwm132* ^*&*^	6B	120	3.9	42.92 ± 1.45	0.297	0.49	112	12.2	47.65 ± 0.87	0.003**	3.9
		Others	96.1	44.86 ± 0.37			Others	87.8	44.38 ± 0.39		
*Xgwm312* ^*#*^	2A	190	28.7	40.03 ± 0.56	0.021*	2.55					
		Others	81.3	38.57 ± 0.35							
*Xgwm372* ^*#*^	2A	331	2	40.40 ± 0.53	0.023*	2.67					
		Others	98	38.74 ± 0.36							
*Xcfa2234* ^*#*^	3A	142	93.5	39.49 ± 0.29	0.002**	4.09					
		Others	6.5	35.95 ± 1.24							
*Xgwm234* ^*#*^	5B	237 and 239	32.2	39.40 ± 0.47	0.737	0.05	227	33.9	40.15 ± 0.53	0.020*	2.43
		Others	67.8	39.19 ± 0.36			Others	66.1	38.78 ± 0.32		
*Xcfd266* ^*#*^	5D	167	33	40.59 ± 0.54	0.001**	4.69	165	54.3	38.96 ± 0.34	0.077	1.42
		Others	67	38.60 ± 0.33			Others	45.7	39.98 ± 0.47		
*Xgwm174* ^*#*^	5D	191	10.9	39.03 ± 0.82	0.951	0	209	20.9	40.33 ± 0.57	0.018*	2.59
		Others	89.1	39.08 ± 0.31			Others	79.1	38.71 ± 0.33		
*Xcfa2257* ^*#*^	7A	129	55.7	39.72 ± 0.32	0.035*	2.05					
		Others	44.3	38.57 ± 0.46							
*Xwmc168* ^*#*^	7A	307	3.9	40.64 ± 1.08	0.347	0.39	305	72.2	39.65 ± 0.34	0.037*	2.01
		Others	96.1	39.26 ± 0.30			Others	27.8	38.21 ± 0.52		
*Xwmc17* ^*#*^	7A	182 and 184	42.2	39.38 ± 0.46	0.649	0.09	180	42.6	39.87 ± 0.38	0.028*	2.17
		Others	57.8	39.11 ± 0.37			Others	57.4	38.63 ± 0.39		

^#^ SSR loci associated with TKW reported by Wang et al. [26].

^&^ SSR loci associated with KNPS reported by Zhang et al. [27].

### Validation of favorable alleles in the DH population

**Fig 4:**
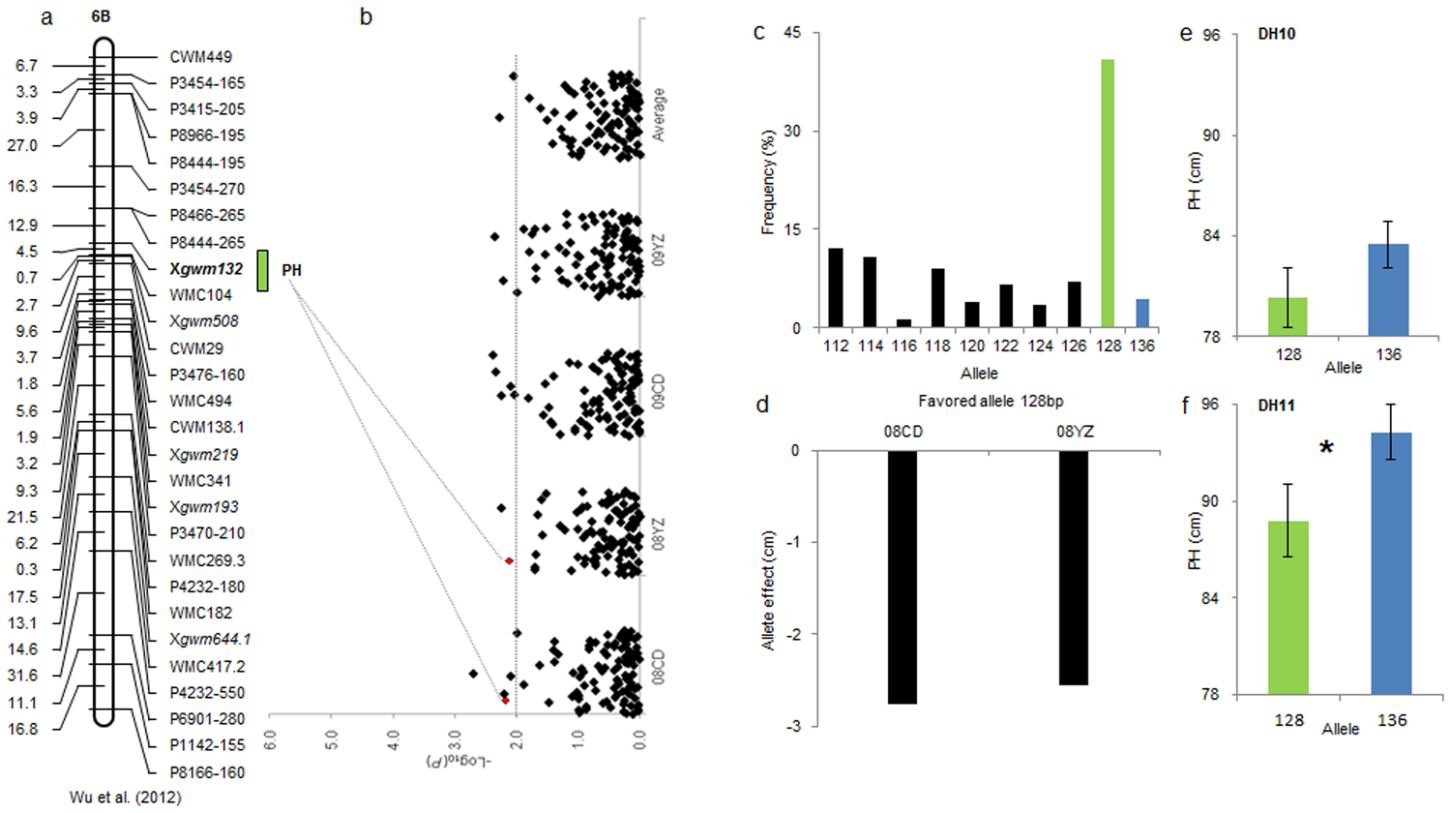
Validation of favorable allele *Xgwm132-6B*_*128*_ on PH in the DH population. a: QTL locus *Xgwm132* for PH on chromosome 6B [7]; b: Associations of PH with 106 SSR markers illustrated as dot plots of compressed MLM at *P*<0.01. Red points represent association signals of *Xgwm132* in different environments; c: Allelic frequenciesfor *Xgwm132* among 230 wheat cultivars, green band represents the 128 bp allele, and blue band represents the 136 bp allele; d: Phenotypic effect of favorable allele *Xgwm132-6B*_*128*_ on PH in the association panel used in this study; e and f: Comparison of average PH values between two alleles in two environments in a DH population. *, significant at *P* = 0.05.

**Fig 5:**
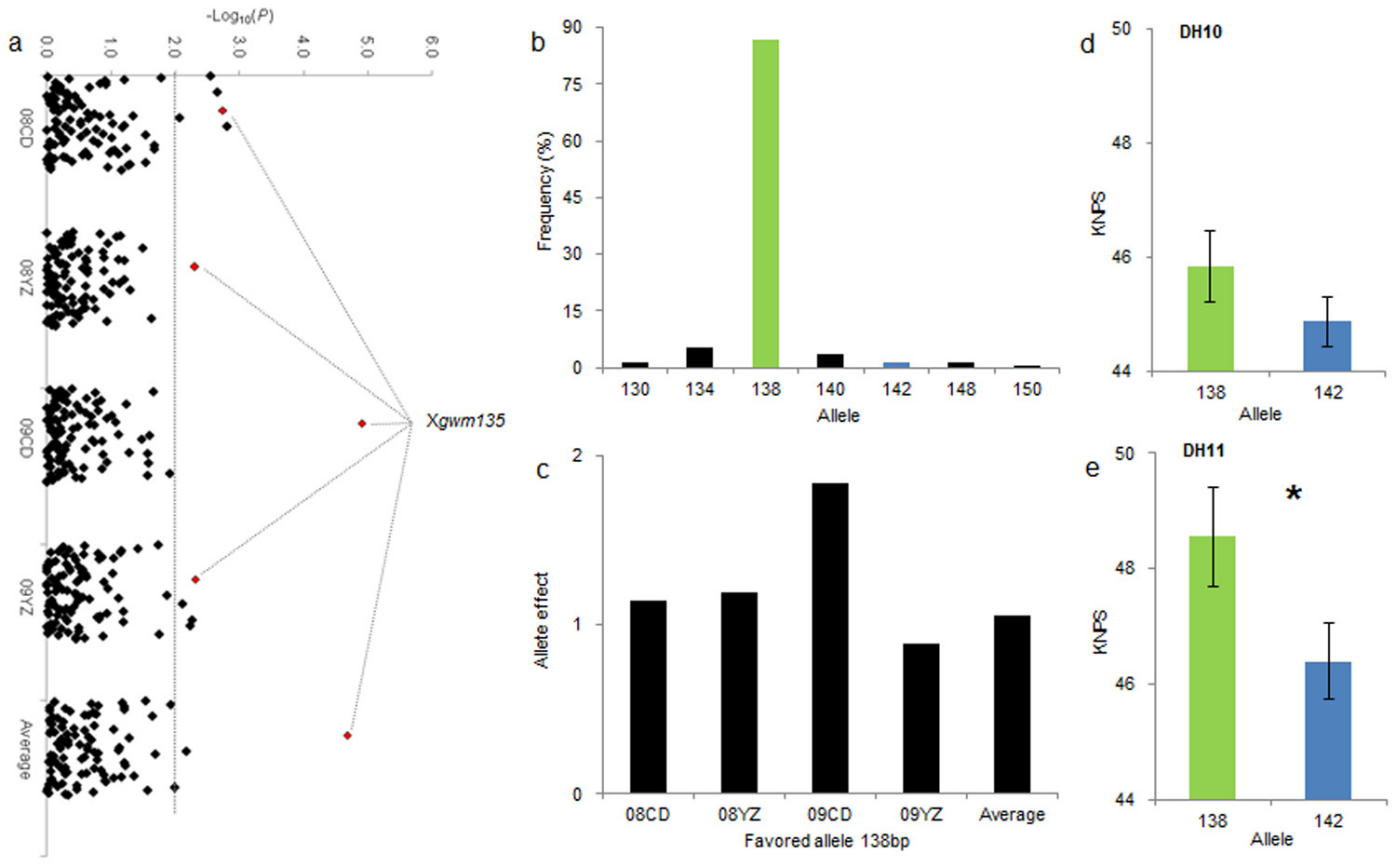
Validation of favorable allele *Xgwm135-1A*_*138*_ on KNPS in the DH population. a: Associations of KNPS with 106 SSR markers illustrated as dot plots of compressed MLM at *P*<0.01. Red points represent association signals of *Xgwm135* in different environments; b: Allelic frequenciesfor *Xgwm135* among 230 wheat cultivars; green band represents the 138 bp allele, and blue band represents the 142 bp allele; c: Phenotypic effects on KNPS of favorable allele *Xgwm135-1A*_*138*_ in the association panel used in this study; d and e: Comparison of average KNPS between two alleles in two environments in the DH population. *,significant at *P* = 0.05.

In order to validate the genetic effects of alleles detected in the association panel, SSR loci significantly associated with yield-related traits were investigated for polymorphism between cultivars Hanxuan10 and Lumai14. A total of 19 SSR were polymorphic between the two cultivars, and were used to genotype the DH population. Statistical comparisons of phenotypic data identified significant differences between alleles at *Xgwm135-1A*, *Xgwm337-1D*, *Xgwm102-2D* and *Xgwm132-6B* in at least one of the two environments in which the DH population was grown (Table 5, Figs 4e, 4f, 5d and 5e). Trait data for favorable alleles were higher than those for pooled ‘other’ categories, although the differences were not statistically significant (Table 5). Therefore, the genetic effects of favorable alleles at these loci were confirmed in both a cultivar association panel and a DH population. *Xgwm132* was linked with a PH QTL in a previous report (Fig 4a) [7]. *Xgwm132* was associated with PH in two environments, and the favorable 128 bp allele had the highest frequency among 10 alleles at this locus (Fig 4b and 4c). The PH effects of *Xgwm132*_*128*_ were -2.76 and -2.56 cm in 08CD and 08YZ, respectively (Fig 4d). This was further verified in the DH population, i.e. -3.20 and -5.50 cm in environments DH10 and DH11, respectively (Fig 4e and 4f). *Xgwm135* was also associated with KNPS in four environments (Fig 5a.) The favorable 138 bp allele occurred at the highest frequency among seven alleles (Fig 5b). The phenotypic effects of *Xgwm135*_*138*_ were 1.14, 1.19, 1.84 and 0.89 in 08CD, 08YZ, 09CD and 09YZ, respectively (Fig 5c). Positive effects of 0.97 and 2.15 on KNPS were also confirmed in DH10 and DH11, respectively (Fig 5d and 5e).

**Table 5:** Favorable alleles and their genetic effects validated in the DH population.

Trait	Locus	Chr.	Environment	Allele (bp)	Freq.(%)	Mean ± SE	Allele effect	*P* value
SNPS	*Xgwm337*	1D	DH10	186	55.56	16.82 ± 0.15	0.43	0.036
				Others	44.44	16.40 ± 0.13		
			DH11	186	55.56	18.73 ± 0.13	0.31	0.080
				Others	44.44	18.43 ± 0.12		
KNPS	*Xgwm135*	1A	DH10	138	43.75	45.84 ± 0.63	0.97	0.192
				Others	56.25	44.87 ± 0.43		
			DH11	138	43.75	48.55 ± 0.86	2.15	0.048
				Others	56.25	46.40 ± 0.67		
	*Xgwm102*	2D	DH10	144	18.88	46.61 ± 0.97	1.44	0.196
				Others	81.12	45.17 ± 0.40		
			DH11	144	18.88	49.84 ± 1.43	3.17	0.019
				Others	81.12	46.67 ± 0.56		
PH	*Xgwm132*	6B	DH10	128	42.76	80.29 ± 1.77	-3.20	0.156
				Others	57.24	83.49 ± 1.41		
			DH11	128	42.76	88.76 ± 2.25	-5.50	0.049
				Others	57.24	94.26 ± 1.70		

## Discussion

### Association analysis is more effective than biparental crosses in identifying yield-related genes

Association mapping and bi-parental population mapping utilize information about genetic recombination and the methods are complementary in identifying genes or QTLs [42]. However, association mapping is more powerful in detecting superior alleles from a large sample of germplasm collections.

In previous studies several SSR loci reported to be associated with yield-related traits (Table 2). *Xwmc24-1A* and *Xgwm132-6B* were associated with QTLs for KNPS [7, 41]; *Xgwm484-2D* with KWPS [19]; *Xgwm155-3A*, *Xgwm186-5A*, *Xgwm132-6B* and *Xgwm46-7B* with PH [7, 10, 25, 43]; *Xgwm182-5D* for SL [44]; *Xgwm297-7B* and *Xgwm186-5A* with SSN [7, 45]; and *Xbarc56-5A* and *Xwmc361-2B* with TKW [45–49]. By association analysis of a panel of cultivar, we not only confirmed earlier marker/trait associations, but also found several new associations of markers and yield-related traits (Tables 2 and 5), such as an association of *Xgwm135-1A* with KNPS in four environments, *Xgwm102-2D* with KNPS, KWPS and SSN, and *Xgwm337-1D* with SNPS. Association mapping combined with bi-parental population analysis is even more powerful in identifying closely linked molecular markers involving yield-related genes [50, 51]. For example, in a QTL analysis of drought tolerance in three RIL populations using SNP markers and 305 diverse inbred lines in maize, Lu et al. [52] found that joint linkage-LD mapping identified 18 QTLs additional to those detected in separate linkage and LD analyses. Korir et al. [53] detected five markers associated with aluminum tolerance in both an association panel of 188 cultivars as well as184 RILs from a bi-parental soybean cross, confirming that these loci should be the best candidate regions to target. Twenty two seed weight and silique length-related QTLs were detected in three bi-parental populations in rapeseed. Among them, *uq*.*A09-1* and *uq*.*A09-3* were identified in all four environments and fine mapped in a set of 576 inbred lines using association analysis [42]. Four associated SSR loci, *Xgwm135-1A*, *Xgwm337-1D*, *Xgwm102-2D* and *Xgwm132-6B*, were detected in our bi-parental population (Table 5, Figs 4e, 4f, 5d and 5e). They had effects on increasing spikelet and kernel numbers and decreasing plant height. These results demonstrate the power of combined association and bi-parental analyses in identifying closely linked molecular markers for economic traits.

### Genetic effects of associated loci were panel-dependent

In previous studies Wang et al. [26] and Zhang et al. [27] used Chinese wheat mini core collection (MCC) to perform association analysis between TKW, KNPS and SSR markers. That collection represented 1% of the national germplasm collection, but more than 70% of the genetic diversity [54]. We genotyped40 SSR loci associated with TKW and KNPS from Wang et al. [26] and Zhang et al. [27]. Only three loci, *Xbarc56-5A* associated with TKW, and *Xwmc24-1A* and *Xgwm132-6B* associated with KNPS showed significant associations. A possible reason for the low number was that the present set comprised released cultivars, among which the allelic profiles were very different. ANOVA showed that 18 of 40 SSR loci had genetic effects on either TKW or KNPS (Table 4). This indicated that genetic effects of loci were also influenced by the population entries. In addition, the *R* ^*2*^ values for TKW at nine loci were much lower than reported for the MCC panel. For example, the earlier *R* ^*2*^ values for *Xcfa2234-3A*_*142*_ and *Xcfa2257-7A*_*129*_ were 18.20 and 21.99%, respectively [26, 27], but were only 4.09 and 2.05% in the present study (Table 4). This lower variation is likely due to the effects of long term selection in breeding programs because the frequencies of *Xcfa2234-3A*_*142*_ and *Xcfa2257-7A*_*129*_ in the earlier reports were 43.9 and 20.6%, respectively [26, 27], but were 93.5 and 55.7% in the current cultivar panel (Table 4). Because released cultivars usually carry superior alleles at crucial loci the genetic effects of those loci were greatly reduced and the *R* ^*2*^ values were lower. For example, Qin et al. [55] detected four haplotypes, *Hap-6B-1*, *Hap-6B-2*, *Hap-6B-3* and *Hap-6B-4*, at *TaGW2-6B*; but the frequencies of *Hap-6B-1* and *Hap-6B-2* showed increasing trends over time, and by the late 1980s *Hap-6B-3* and *Hap-6B-4* had disappeared. The allelic difference between *Hap-6B-1* and *Hap-6B-2* was much smaller than that involving either of them with *Hap-6B-3* or *Hap-6B-4*. Therefore, the more important a locus is for an agronomic trait, the stronger it will be selected in breeding. Hence the *R* ^*2*^ value should decline from a random germplasm collection to a released cultivar population.

Genetic effects were also affected by environment (G x E). Flowering time in maize is a complex trait affected by genes and the environments. *ZmCCT* is one of the most important genes affecting photoperiod response. Hung et al. [56] found that many maize inbred lines carried *ZmCCT* alleles with no sensitivity to day length, allowing breeders to produce more widely adapted maize varieties. In the current study, association signals were detected in multiple-environments, i.e. 08CD, 09CD, 08YZ and 09YZ. Average values of yield-related traits were also calculated according to the BLUP method and associated with SSRs. Based on association detection using BLUP mean values, seven SSR loci had significant association signals in two or more environments, such as *Xgwm135-1A* with KNPS, *Xgwm515-2A* and *Xgwm132-6B* with PH, *Xgwm219-6B* with SNPS, and *Xgwm102-2D*, *Xgwm297-7B* and *Xgwm383-3D* with SSN. Therefore, the influence of environments on genetic effects of the associated loci was indirectly reflected by comparison of their values in different environments. Hence, there was a higher influence if an associated locus had an effect in only one or few environments (Table 3).

### Favorable alleles in past and future wheat breeding

Some loci have played important roles in wheat breeding. A good example is *Xgwm261* that is 0.6 cM from *Rht8* on chromosome 2D. The favorable *Xgwm261* allele (192 bp) is associated with an approximate 10 cm reduction in plant height [3]. *Rht8* is also closely linked with *Ppd-D1*, which affects varietal adaptability leading to increased grain yield in certain environments [4]. Zhou et al. [57] identified *Rht8* in many Chinese wheat varieties widely grown in the last 30 years. About 40% of varieties contained *Rht8* based on pedigree, but its frequency varied in different ecological zones. Italian cultivars Funo, Villa Glory, St1472/506 and St2422/464, widely used as founder genotypes in Chinese wheat breeding [58, 59], are all carriers of *Rht8*. Grain weight also underwent strong selection during wheat breeding. For example, *TaGW*2-*A1* significantly associated with TGW [60] was mapped to chromosome 6A. The superior haplotype *Hap-6A-A* increases TGW by more than 3 g. Among loci that were validated in the DH population in this study the frequencies of favorable alleles *Xgwm135-1A*_*138*_ and *Xgwm102-2D*_*144*_ with positive effects on KNPS exceeded 50%, suggesting that these loci have contributed to Chinese wheat breeding (Tables 3 and 5). On the other hand, the frequency of *Xgwm337-1D*_*186*_ with a clear genetic effect verified in both populations was only 5.22%. Clearly that allele could be selected to increase SNPS in future marker-assisted selection (MAS) breeding. Thus, strong selection in the breeding of newly released cultivars has already focused on some favorable alleles [26, 27, 61]. Modern Chinese varieties produced over the last 60 years are based on 16 founder parents [59]. Some of these founder parents were included in the 230 wheat cultivars used in this study. Abbondanza, Funo, and St2422/464, for example, carried more favorable alleles than some other founder parents in our study as was also reported by Ge et al. [61].

In summary, four favorable alleles, namely, *Xgwm135-1A*_*138*_, *Xgwm337-1D*_*186*_, *Xgwm102-2D*_*144*_, and *Xgwm132-6B*_*128*_, identified in this study will be useful in future breeding for high-yield.

## Supporting Information

S1 Table The 230 wheat accessions used in association analysis. (XLSX)

S2 Table The 106 SSR loci used in association analysis. (XLSX)

S3 Table Allele number, MAF and PIC of 106 polymorphic SSR markers detected in the association panel. (DOCX)
