# Supplementary material for: Association and Validation of Yield-Favored Alleles in Chinese Cultivars of Common Wheat (Triticumaestivum L.)
Source: PLoS One. 2015 Jun 11;10(6):e0130029. doi: 10.1371/journal.pone.0130029 (PMC4466017; doi:10.1371/journal.pone.0130029)
Supplement: S3 Table — (DOCX) [file pone.0130029.s003.docx]

**S3 Table Allele number, MAF and PIC of 106 polymorphic SSR markers detected in the association panel**

| Locus | Chr. | MAF | No. of alleles | Genetic diversity | PIC |  | Locus | Chr. | MAF | No of alleles | Genetic diversity | PIC |
| --- | --- | --- | --- | --- | --- | --- | --- | --- | --- | --- | --- | --- |
| *Xwmc24*^&^ | 1A | 0.348 | 7 | 0.748 | 0.707 |  | *Xgwm161* | 3D | 0.381 | 11 | 0.711 | 0.661 |
| *Xcfa2153*^#^ | 1A | 0.583 | 13 | 0.628 | 0.605 |  | *Xgwm383* | 3D | 0.430 | 8 | 0.634 | 0.565 |
| *Xgwm135* | 1A | 0.867 | 7 | 0.244 | 0.237 |  | *Xgwm645* | 3D | 0.233 | 15 | 0.856 | 0.840 |
| *Xgwm164* | 1A | 0.282 | 10 | 0.759 | 0.718 |  | *Xgwm160* | 4A | 0.332 | 7 | 0.752 | 0.713 |
| *Xgwm357* | 1A | 0.719 | 4 | 0.447 | 0.410 |  | *Xgwm610* | 4A | 0.399 | 5 | 0.693 | 0.638 |
| *Xgwm259*^&^ | 1B | 0.417 | 7 | 0.731 | 0.693 |  | *Xgwm149* | 4B | 0.779 | 8 | 0.378 | 0.360 |
| *Xgwm403*^#^ | 1B | 0.697 | 7 | 0.476 | 0.439 |  | *Xgwm495* | 4B | 0.386 | 11 | 0.777 | 0.751 |
| *Xgwm268*^#^ | 1B | 0.164 | 19 | 0.906 | 0.899 |  | *Xgwm513* | 4B | 0.518 | 5 | 0.636 | 0.582 |
| *Xgwm124* | 1B | 0.930 | 6 | 0.134 | 0.130 |  | *Xgwm538* | 4B | 0.895 | 3 | 0.190 | 0.175 |
| *Xgwm413* | 1B | 0.330 | 16 | 0.787 | 0.758 |  | *Xcfd106*^&^ | 4D | 0.987 | 3 | 0.026 | 0.026 |
| *Xgwm498* | 1B | 0.681 | 2 | 0.434 | 0.340 |  | *Xgwm609*^&^ | 4D | 0.505 | 13 | 0.705 | 0.683 |
| *Xgwm337*^&^ | 1D | 0.457 | 11 | 0.751 | 0.733 |  | *Xgwm194* | 4D | 0.527 | 6 | 0.611 | 0.544 |
| *Xwmc147*^#^ | 1D | 0.719 | 5 | 0.423 | 0.360 |  | *Xbarc56*^#^ | 5A | 0.507 | 7 | 0.542 | 0.439 |
| *Xgwm232* | 1D | 0.856 | 6 | 0.255 | 0.238 |  | *Xgwm126* | 5A | 0.491 | 5 | 0.586 | 0.501 |
| *Xgwm458* | 1D | 0.764 | 4 | 0.384 | 0.346 |  | *Xgwm186* | 5A | 0.548 | 14 | 0.662 | 0.638 |
| *Xgwm642* | 1D | 0.458 | 7 | 0.642 | 0.578 |  | *Xgwm304* | 5A | 0.464 | 10 | 0.680 | 0.633 |
| *Xgwm249*^&^ | 2A | 0.956 | 4 | 0.086 | 0.084 |  | *Xgwm415* | 5A | 0.571 | 2 | 0.490 | 0.370 |
| *Xgwm311*^&^ | 2A | 0.802 | 5 | 0.343 | 0.325 |  | *Xgwm234*^#^ | 5B | 0.344 | 12 | 0.807 | 0.786 |
| *Xgwm275*^#^ | 2A | 0.333 | 9 | 0.771 | 0.736 |  | *Xwmc415*^#^ | 5B | 0.695 | 6 | 0.471 | 0.425 |
| *Xgwm312*^#^ | 2A | 0.295 | 16 | 0.817 | 0.794 |  | *Xgwm499* | 5B | 0.697 | 13 | 0.490 | 0.466 |
| *Xgwm372*^#^ | 2A | 0.210 | 22 | 0.872 | 0.859 |  | *Xgwm540* | 5B | 0.607 | 7 | 0.599 | 0.574 |
| *Xgwm95* | 2A | 0.478 | 9 | 0.626 | 0.558 |  | *Xcfd52*^&^ | 5D | 0.511 | 5 | 0.547 | 0.446 |
| *Xgwm294* | 2A | 0.263 | 14 | 0.824 | 0.802 |  | *Xbarc322*^&^ | 5D | 0.480 | 6 | 0.662 | 0.608 |
| *Xgwm512* | 2A | 0.722 | 3 | 0.404 | 0.326 |  | *Xcfd266*^#^ | 5D | 0.551 | 5 | 0.580 | 0.507 |
| *Xgwm515* | 2A | 0.498 | 6 | 0.607 | 0.532 |  | *Xgwm174*^#^ | 5D | 0.218 | 25 | 0.908 | 0.903 |
| *Xgwm526*^&^ | 2B | 0.473 | 6 | 0.692 | 0.652 |  | *Xgwm182* | 5D | 0.732 | 6 | 0.436 | 0.406 |
| *Xwmc361*^&^ | 2B | 0.756 | 5 | 0.387 | 0.338 |  | *Xgwm190* | 5D | 0.665 | 6 | 0.516 | 0.478 |
| Locus | Chr. | MAF | No. of alleles | Genetic diversity | PIC |  | Locus | Chr. | MAF | No of alleles | Genetic diversity | PIC |
| *Xgwm120* | 2B | 0.464 | 17 | 0.750 | 0.734 |  | *Xgwm212* | 5D | 0.872 | 4 | 0.229 | 0.214 |
| *Xgwm148* | 2B | 0.386 | 7 | 0.726 | 0.683 |  | *Xgwm292* | 5D | 0.696 | 10 | 0.493 | 0.471 |
| *Xgwm374* | 2B | 0.629 | 5 | 0.557 | 0.518 |  | *Xgwm358* | 5D | 0.757 | 4 | 0.400 | 0.369 |
| *Xgwm429* | 2B | 0.422 | 12 | 0.732 | 0.697 |  | *Xgwm583* | 5D | 0.408 | 6 | 0.678 | 0.617 |
| *Xgwm501* | 2B | 0.392 | 12 | 0.776 | 0.752 |  | *Xcfe273*^&^ | 6A | 0.573 | 2 | 0.489 | 0.370 |
| *Xgwm102* | 2D | 0.500 | 8 | 0.613 | 0.544 |  | *Xgwm82* | 6A | 0.805 | 4 | 0.332 | 0.306 |
| *Xgwm261* | 2D | 0.553 | 8 | 0.612 | 0.560 |  | *Xgwm169* | 6A | 0.617 | 11 | 0.590 | 0.567 |
| *Xgwm484* | 2D | 0.283 | 21 | 0.868 | 0.857 |  | *Xgwm570* | 6A | 0.485 | 9 | 0.685 | 0.645 |
| *Xgwm539* | 2D | 0.349 | 18 | 0.800 | 0.779 |  | *Xgwm132*^&^ | 6B | 0.410 | 10 | 0.782 | 0.764 |
| *Xgwm2*^&^ | 3A | 0.727 | 6 | 0.443 | 0.413 |  | *Xgwm193* | 6B | 0.729 | 7 | 0.425 | 0.379 |
| *Xcfa2234*^#^ | 3A | 0.935 | 2 | 0.122 | 0.114 |  | *Xgwm219* | 6B | 0.404 | 12 | 0.709 | 0.667 |
| *Xgwm5* | 3A | 0.580 | 9 | 0.560 | 0.491 |  | *Xgdm127*^&^ | 6D | 0.852 | 3 | 0.263 | 0.247 |
| *Xgwm155* | 3A | 0.356 | 10 | 0.752 | 0.715 |  | *Xgwm55*^#^ | 6D | 0.345 | 11 | 0.819 | 0.801 |
| *Xgwm218* | 3A | 0.554 | 9 | 0.654 | 0.629 |  | *Xgwm469* | 6D | 0.438 | 11 | 0.760 | 0.740 |
| *Xgwm131*^&^ | 3B | 0.400 | 4 | 0.660 | 0.588 |  | *Xgwm471*^#^ | 7A | 0.358 | 22 | 0.834 | 0.824 |
| *Xgwm108*^&^ | 3B | 0.535 | 6 | 0.610 | 0.546 |  | *Xwmc168*^#^ | 7A | 0.735 | 10 | 0.446 | 0.429 |
| *Xgwm156*^#^ | 3B | 0.348 | 13 | 0.787 | 0.760 |  | *Xwmc17*^#^ | 7A | 0.426 | 6 | 0.643 | 0.573 |
| *Xgwm247* | 3B | 0.300 | 13 | 0.775 | 0.741 |  | *Xcfa2257*^#^ | 7A | 0.569 | 2 | 0.491 | 0.370 |
| *Xgwm285* | 3B | 0.617 | 14 | 0.599 | 0.583 |  | *Xgwm130* | 7A | 0.476 | 9 | 0.673 | 0.624 |
| *Xgwm389* | 3B | 0.533 | 11 | 0.683 | 0.663 |  | *Xgwm297* | 7B | 0.391 | 11 | 0.756 | 0.724 |
| *Xgwm2*^&^ | 3D | 0.726 | 6 | 0.445 | 0.414 |  | *Xgwm333* | 7B | 0.590 | 5 | 0.597 | 0.557 |
| *Xcfd64*^&^ | 3D | 0.856 | 4 | 0.253 | 0.232 |  | *Xgwm537* | 7B | 0.463 | 10 | 0.724 | 0.695 |
| *Xbarc42*^&^ | 3D | 0.691 | 3 | 0.432 | 0.346 |  | *Xgwm46* | 7B | 0.279 | 16 | 0.852 | 0.838 |
| *Xcfe172*^&^ | 3D | 0.887 | 4 | 0.180 | 0.172 |  | *Xbarc258* | 7B | 0.493 | 5 | 0.660 | 0.608 |
| *Xgwm3* | 3D | 0.399 | 6 | 0.727 | 0.683 |  | *Xgwm44* | 7D | 0.288 | 11 | 0.829 | 0.810 |
| *Xgwm52* | 3D | 0.569 | 7 | 0.612 | 0.569 |  | *Xgwm428* | 7D | 0.469 | 7 | 0.684 | 0.637 |

^#^ SSR loci associated with TKW reported by Wang et al. [26].

^&^ SSR loci associated with KNPS reported by Zhang et al. [27].
